# Metal-Free Restorations: Esthetic Considerations, Treatment Planning, Preparation, Manufacturing, Luting, and Followup

**DOI:** 10.1155/2015/382418

**Published:** 2015-12-17

**Authors:** Hamit S. Çötert, Handan Yılmaz, Cem Kurtoğlu, Flávio H. B. Aguiar, Ahmet U. Güler

**Affiliations:** ^1^Department of Prosthodontics, Faculty of Dentistry, Ege University, 35100 Izmir, Turkey; ^2^Department of Prosthodontics, Faculty of Dentistry, Gazi University, 06500 Ankara, Turkey; ^3^Department of Prosthodontics, Faculty of Dentistry, Çukurova University, 01330 Adana, Turkey; ^4^Department of Restorative Dentistry, Piracicaba Dental School, University of Campinas, 13414-093 Piracicaba, SP, Brazil; ^5^Medical Park Bahçelievler Hospital, Bahçelievler, 34160 İstanbul, Turkey

The science and art of dental medicine are progressing with a vertiginous speed. Restorative and prosthetic dentistry can be considered as the fastest growing branches due to their close interactions with material science, chemistry, engineering, and computer science. Only two decades ago did dental practitioners had corrected the unsightly appearance of their patients' teeth by using full-coverage crown restorations. Ceramometal combinations were the material of choice for such restorations. In spite of the fact that these crowns were quite esthetic, resistant, and long lasting, they were still requiring invasive tooth preparation techniques. After the improvements in resin composite restorative materials and ceramics and manufacturing, bonding, and luting techniques and improvements in the preoperative design approaches, most of the dental operators have substituted the ceramometal full-coverage crowns with metal-free full or partial-coverage restorations made from resin composites or ceramics. Honestly, initial signs of these improvements have been introduced earlier, in the second half of the last century. These initial signs were the acid etching of the tooth enamel described by Buonocore and the chemical composition of the Bis-GMA introduced by Bowen. During the nineties, the velocity of the scientific investigations and researches shifted to describe, progress, compare, and observe the several restorative materials, manufacturing techniques, bonding, and/or luting agents and methods. Performed restorations have also been collected as case series, followed, analyzed, and reported. Nevertheless, the magnificent enlargement and the growing speed of the cumulative data subjecting the metal-free restorations are required to be reviewed and upgraded as frequent as possible. For this purpose the present special issue has been conducted to collect the most recent research reports that subjected the esthetic considerations, treatment planning, tooth preparation, manufacturing, luting, and the followup of the metal-free restorations.

Ten precious articles written by fifty authors from all over the world have been included in this special issue. The recent knowledge about the surface treatment methods of the hard tooth tissues as well as the glass-ceramics and zirconia; marginal fit and surface finishing of CAD-CAM fabricated monolithic and bilayered zirconia crowns; surface characteristics of the direct resin composite restoratives after bleaching are discussed. A meticulous literature review objecting glass-ceramic crowns is also included in order to help the readers to refresh their knowledge.

C. R. G. van den Breemer et al. systematically organized the current knowledge regarding the cementation of glass-ceramic materials and restorations in a comprehensive review. They additionally discussed the benefits of Immediate Dentin Sealing (IDS). D. P. Lameira et al. evaluated the effect of design and surface finishing on fracture strength of monolithic and bilayered zirconia crowns after artificial aging. K.-H. Lee et al. investigated the marginal fit of metal-free crowns made by three different computer-aided design/computer-aided manufacturing (CAD/CAM) systems and G. Pompa et al. compared the marginal fit of the zirconia crowns with the laser sintered and cast metal crowns. Y. Lee et al. analyzed the microshear bond strength of self-adhesive resin cement on leucite-reinforced glass-ceramic, with or without surface treatment using pure silane or a universal adhesive. J.-S. Ahn et al. evaluated the effects of different phosphate monomer-containing primers on the shear bond strength between zirconia and MDP-containing self-adhesive resin cement. M. Piemjai and N. Nakabayashi reported the tensile strength and characteristics of dentin restored with all-ceramic, resin composite and cast metal prostheses cemented with resin adhesives. C. Bernard et al. assessed the effect of radiotherapy on bond efficiency of two different adhesive systems. R. J.-Y. Kim et al. observed the temperature changes at various sites within the composite and on the pulpal side of dentin during polymerization of composite increments. B. A. Irawan et al. exhibited the effects of the vital tooth bleaching on the color stability and 3D surface profile of dental restorative filling materials.

*Hamit S. Çötert* *Handan Yılmaz* *Cem Kurtoğlu* *Flávio H. B. Aguiar* *Ahmet U. Güler*

